# Supplementary material for: Efficient CRISPR/Cas9-based genome editing and its application to conditional genetic analysis in Marchantia polymorpha
Source: PLoS One. 2018 Oct 31;13(10):e0205117. doi: 10.1371/journal.pone.0205117 (PMC6209168; doi:10.1371/journal.pone.0205117)
Supplement: S1 Fig — (A) pMpGE_En01 is designed to use the In-Fusion/Gibson cloning methods. pMpGE_En01 is digested by SacI and PstI. Two entirely complementary oligo DNAs, which contain at both ends the 15-bp sequences identical to each end of the digested vector and a guide sequence without PAM sequence in between, are annealed and cloned by use the In-Fusion/Gibson reaction. The sense strand of gRNAs should be coded in oligo F. The ‘extra initial G’ is colored in magenta. (B) pMpGE_En02/03 are designed to use ligation reactions. pMpGE_En02 or pMpGE_En03 is digested by BsaI, which digests outside of its recognition sites. Oligo F, which contains a sense-strand guide sequence with TCTC at its 5’ end, and oligo R, which contains the reverse-complement guide sequence with AAAC at its 5’ end, are annealed and cloned by ligation reaction to pMpGE_En02. pMpGE_En02 does not contain an ‘extra initial G’ and thus requires G or A in the first nucleotide position of a guide sequence for efficient expression. In case of pMpGE_En03, the ‘extra initial G’ exists in the vector. Therefore, oligo F should contain a sense-strand guide sequence with CTCG at its 5’ end for the construction of pMpGE_En03. (PDF) [file pone.0205117.s001.pdf]

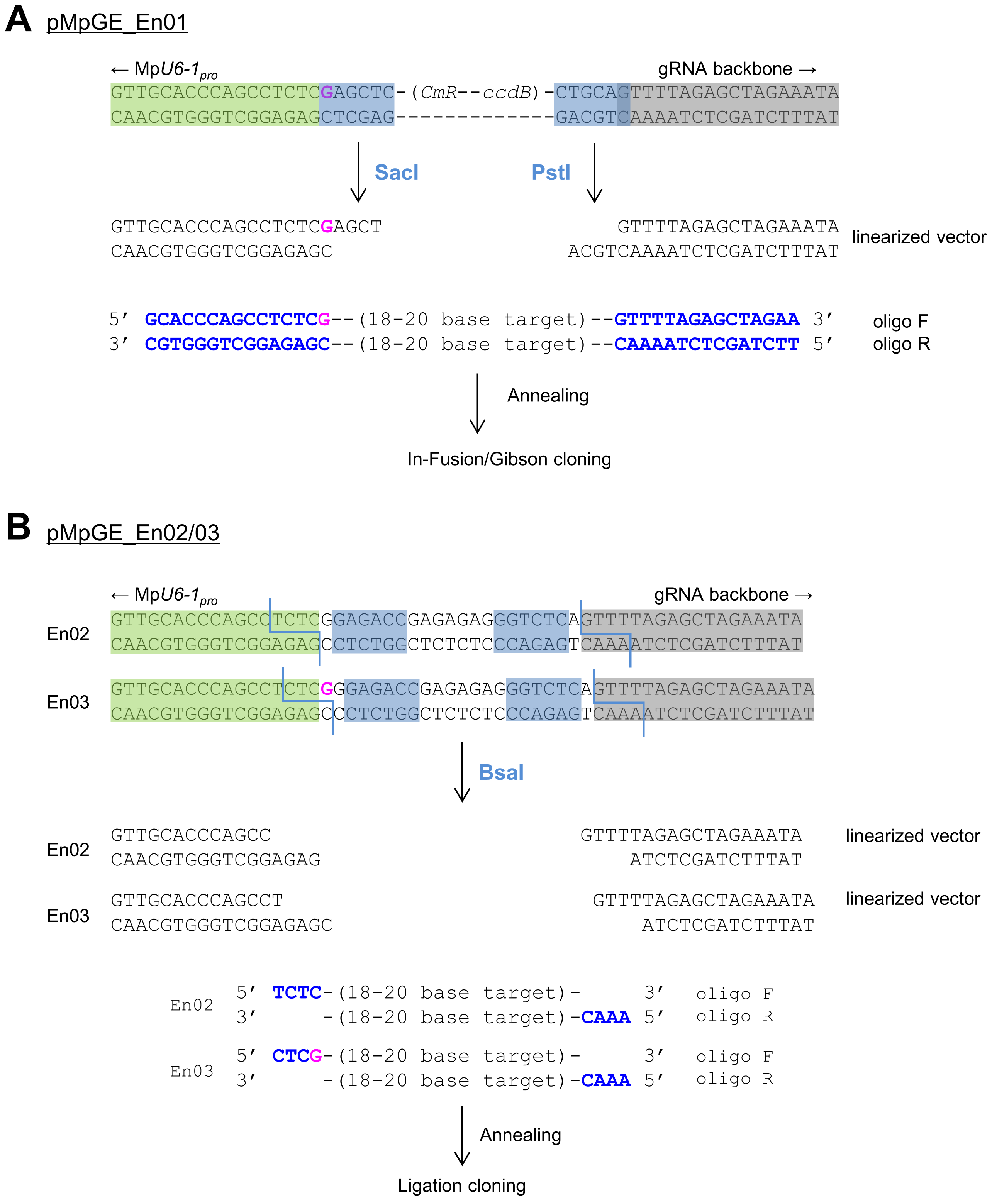
A
pMpGE_En01
← MpU6-1pro
gRNA backbone →
GTTGCACCCAGCCTCTCGAGCTC-(CmR--ccdB)-CTGCAGTTTTAGAGCTAGAAATA
CAACGTGGGTCGGAGAGCTCGAG-------------GACGTCAAAATCTCGATCTTTAT
SacI
PstI
GTTGCACCCAGCCTCTCGAGCT
CAACGTGGGTCGGAGAGC
GTTTTAGAGCTAGAAATA
ACGTCAAAATCTCGATCTTTAT
linearized vector
5′ GCACCCAGCCTCTCG--(18-20 base target)--GTTTTAGAGCTAGAA 3′
3′ CGTGGGTCGGAGAGC--(18-20 base target)--CAAAATCTCGATCTT 5′
oligo F
oligo R
Annealing
In-Fusion/Gibson cloning
B
pMpGE_En02/03
← MpU6-1pro
gRNA backbone →
En02
GTTGCACCCAGCCTCTCGGAGACCGAGAGAGGGTCTCAGTTTTAGAGCTAGAAATA
CAACGTGGGTCGGAGAGCCTCTGGCTCTCTCCCAGAGTCAAAATCTCGATCTTTAT
En03
GTTGCACCCAGCCTCTCGGGAGACCGAGAGAGGGTCTCAGTTTTAGAGCTAGAAATA
CAACGTGGGTCGGAGAGCCCTCTGGCTCTCTCCCAGAGTCAAAATCTCGATCTTTAT
BsaI
En02
GTTGCACCCAGCC
CAACGTGGGTCGGAGAG
GTTTTAGAGCTAGAAATA
ATCTCGATCTTTAT
linearized vector
En03
GTTGCACCCAGCCT
CAACGTGGGTCGGAGAGC
GTTTTAGAGCTAGAAATA
ATCTCGATCTTTAT
linearized vector
En02
5′ TCTC-(18-20 base target)- 3′
3′ -(18-20 base target)-CAAA 5′
oligo F
oligo R
En03
5′ CTCG-(18-20 base target)- 3′
3′ -(18-20 base target)-CAAA 5′
oligo F
oligo R
Annealing
Ligation cloning
