# Supplementary figures and images for: Efficient CRISPR/Cas9-based genome editing and its application to conditional genetic analysis in Marchantia polymorpha
Source: PLoS One. 2018 Oct 31;13(10):e0205117. doi: 10.1371/journal.pone.0205117 (PMC6209168; doi:10.1371/journal.pone.0205117)

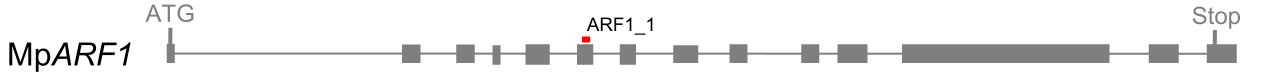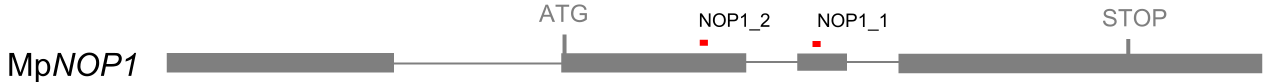

Supplement: S2 Fig — Target sites of the gRNAs used are shown as red lines. Boxes and lines show exons and introns, respectively. “ATG” and “Stop” denote the predicated initiation and termination codons. (PDF) [file pone.0205117.s002.pdf]

## hCas9

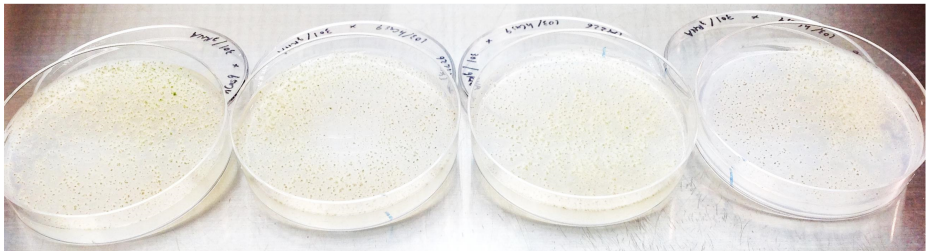

## Atco-Cas9

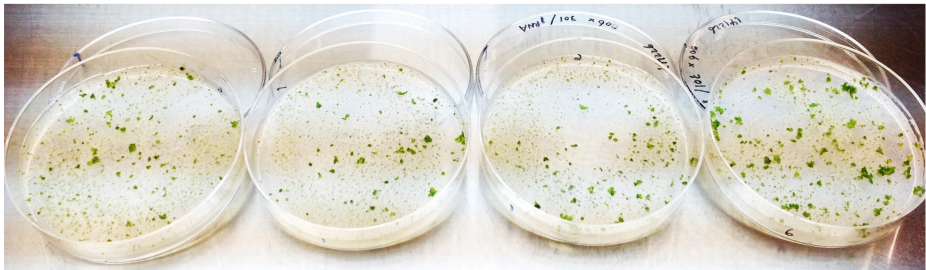

10  $\mu$ M NAA, 3 weeks

Supplement: S3 Fig — The same MpARF1-trageting gRNA expression vector (pMpGWB301_ARF1_1) was introduced into sporelings together with either hCas9 expression vector (pMpGWB103-hCas9; top) or Atco-Cas9 expression vector (pMpGE006; bottom) and selected on media containing 10 μM NAA for three weeks. Mutations in the MpARF1 gene are known to cause NAA resistance. (PDF) [file pone.0205117.s003.pdf]

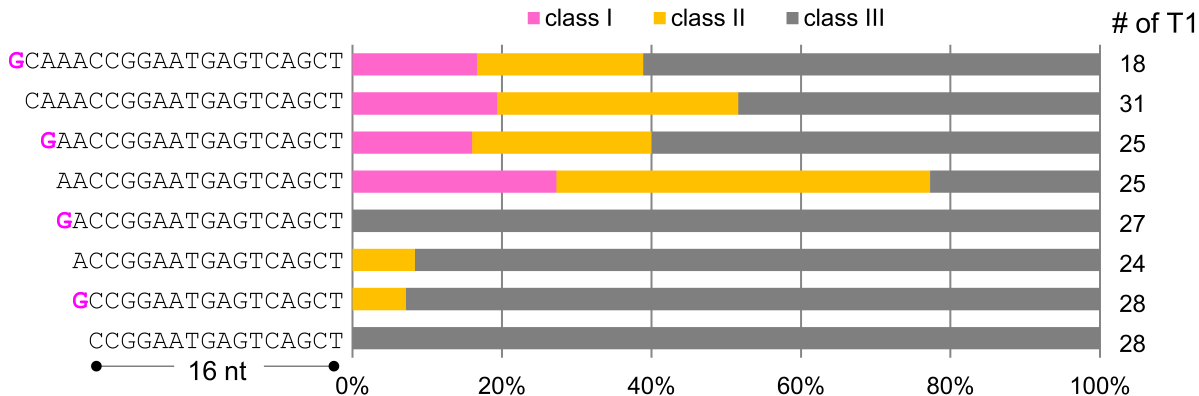

Supplement: S5 Fig — Proportions of mutant phenotype classes (see Fig 4) in T1 plants transformed with MpNOP1-targeting gRNAs (NOP1_2) of the indicated lengths with or without the ‘extra initial G’ (magenta). The numbers of T1 plants inspected are shown on the right side of the graph. (PDF) [file pone.0205117.s005.pdf]

Tak-1

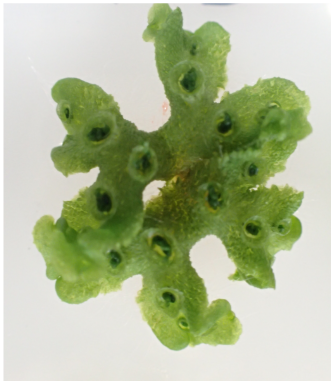

*Mpnop1<sup>ge</sup>*

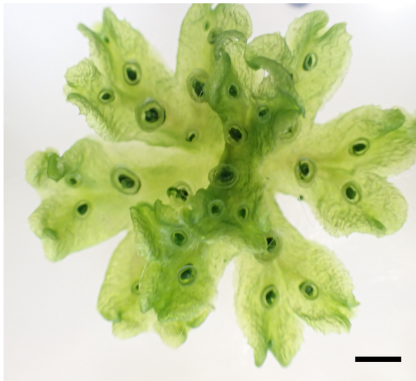

Supplement: S6 Fig — Photos of Tak-1 control plant (left) and the double transformant, which pMpGE013 with NOP1_3 gRNA and pMpGE014 with NOP1_6 gRNA were transfected (right). Scale bar = 2 mm. (PDF) [file pone.0205117.s006.pdf]

**A**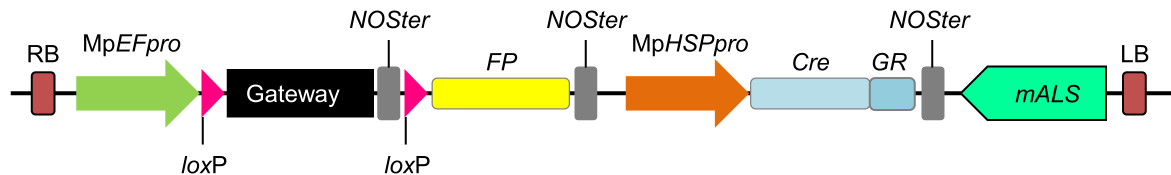**B**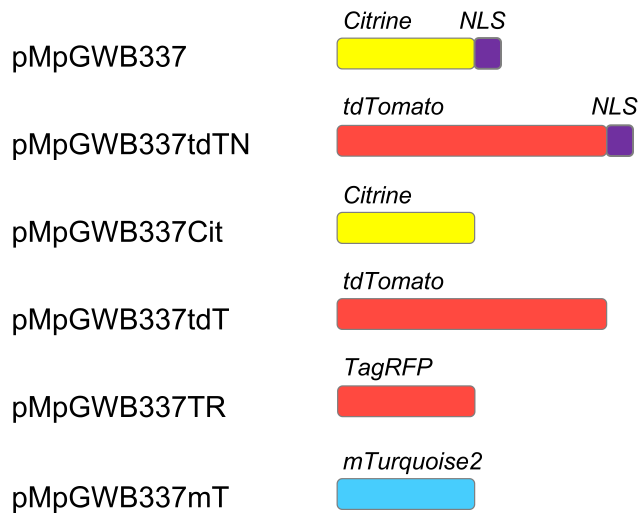

Supplement: S7 Fig — (A) Structure of pMpGWB337 derivatives. Genes for complementation (either cDNA or genomic fragment) can be expressed under the control of MpEFpro by introduction into the Gateway cassette and deleted in plants by heat shock and DEX treatment by virtue of the cassette expressing Cre recombinase fused to the rat glucocorticoid receptor domain (GR) under the control of the MpHSP17.8A1 promoter [23]. FP, fluorescent protein coding sequence. (B) List of fluorescent protein sequences in pMpGWB337 derivatives. NLS, nuclear localization signal. Note: These vectors can be used for the conditional knockout experiment in combination with the pMpGE series (Fig 7). In this strategy, the complementation gene cassette must have a structure that cannot be targeted by the gRNA used for knocking out the target gene. This “gRNA-resistant” complementation cassette can be prepared by introducing synonymous substitutions in the matching sequence. Alternatively, if a gRNA can be designed at exon-intron junctions in such a way shown in Fig 7, a non-modified cDNA can be readily used. A DNA fragment for complementation can be inserted between the two loxP sites in the vectors by using the Gateway technology. These all-in-one vectors are equipped with a floxed Gateway cassette and with a heat-shock- and DEX-inducible Cre recombinase expression cassette and lined up with various fluorescent protein markers. (PDF) [file pone.0205117.s007.pdf]
