# Supplementary material for: Efficient CRISPR/Cas9-based genome editing and its application to conditional genetic analysis in Marchantia polymorpha
Source: PLoS One. 2018 Oct 31;13(10):e0205117. doi: 10.1371/journal.pone.0205117 (PMC6209168; doi:10.1371/journal.pone.0205117)
Supplement: S4 Fig — (A) MpARF1-targeted mutagenesis with pMpGE011 containing the gRNA expression cassette for ARF1_1 derived from pMpGE_En01. (B) MpNOP1-targeted mutagenesis with MpGE010 containing the gRNA expression cassette for NOP1_1 derived from pMpGE_En02. Inserted or substituted bases are colored in magenta. The target guide sequences are shown in bold face with their PAM sequences in blue. (PDF) [file pone.0205117.s004.pdf]

A

pMpGE011_ARF1_1 using pMpGE_En01 (28/32)

```
    H  P  A Q  E  L  V  A  R  D  L  H  D  Q     E  W  H  F  R
5'-CATCCTGCCCAGGAGCTTGTCGCAAGAGACCTTCATGATCA--GGAGTGGCACTTCCGG-3' (WT)
5'-CATCCTGCCCAGGAGCTTGTCGCAAGAGACCTTCAT--------GAGTGGCACTTCCGG-3'  x2
5'-CATCCTGCCCAGGAGCTTGTCGCAAGAGACCTTCATGAT-----GAGTGGCACTTCCGG-3'  x2
5'-CATCCTGCCCAGGAGCTTGTCGCAAGAGACCTTCATGATCAGTGGAGTGGCACTTCCGG-3'
5'-CATCCTGCCCAGGAGCTTGTCGCAAGAGACCTTCATGATC-------TGGCACTTCCGG-3'
5'-CATCCTGCCCAGGAGCTTGTCGCAAGTGACCTTCATGATCG--TGAGTGGCACTTCCGG-3'
5'-CATCCTGCCCAGGAGCTTGTCGCAAGAGACCTTCATGATGA--GGAGTGGCACTTCCGG-3'  x2
5'-CATCCTGCCCAGGAGCTTGTCGCAAGAGACCTTCATGATCA--TGAGTGGCACTTCCGG-3'  x8
5'-CATCCTGCCCAGGAGCTTGTCGCAAGAGACCTTCATGATCA---GAGTGGCACTTCCGG-3'
5'-CATCCTGCCCAGGAGCTTGTCGCAAGAGACCTTCATGATCT--TGAGTGGCACTTCCGG-3'
5'-CATCCTGCCCAGGAGCTTGTCGCAAGAGACCTTCATGAT-----------CACTTCCGG-3'
Indels larger than 20 bp x 2
Mosaic x 6
```

B

pMpGE010_NOP1_1 using pMpGE_En02 (12/16)

```
   E  L  S  K  S  D  S  L  C  E  R        I  G  S  T  N  G  A
5'-GAGCTCTCGAAATCCGATAGTCTTTGTGAGAG-------AATAGGCTCAACAAACGGAGCTA-3' (WT)
5'-GAGCTCTCGAAATCCGATAGTCTTTGTGAGAG--------CTAGGCTCAACAAACGGAGCTA-3'
5'-GAGCTCTCGAAATCCGATAGTCTTTGTGAGAGGCTCAACAATAGGCTCAACAAACGGAGCTA-3'
5'-GAGCTCTCGAAATCCGATAGTCTTTGTGA------------TAGGCTCAACAAACGGAGCTA-3'
5'-GAGCTCTCGAAATCCGATAGTCTTTGTAA------------TAGGCTCAACAAACGGAGCTA-3'
5'-GAGCTCTCGAAATCCGATAGTCTTTGTGA------------GAGGCTCAACAAACGGAGCTA-3'
5'-GAGCTCTCGAAATCCGATAGTCTTTGTGA---------------------------GAGCTA-3'
5'-GAGCTCTCGAAATCCGATAGTCTTTGTGAGAG-------AATAGGCTCAACAAACGGAGCTA-3'  x4
Indels larger than 20 bp x 4
Mosaic x 2
```
