# Supplementary material for: Efficient CRISPR/Cas9-based genome editing and its application to conditional genetic analysis in Marchantia polymorpha
Source: PLoS One. 2018 Oct 31;13(10):e0205117. doi: 10.1371/journal.pone.0205117 (PMC6209168; doi:10.1371/journal.pone.0205117)
Supplement: S8 Fig — Sporelings were transformed with pMpGE010 harboring ARF1_1 gRNA (Atco-Cas9-NLS) or a combination of pMpGWB103-hCas9-NLS and pMpGWB301_ARF1_1 (hCas9-NLS) and incubated on the selective media for 2 weeks. Obtained ~100 small transformants were collected and used for protein extraction with 1×SDS sample buffer. Extracted protein solutions were diluted by 2 folds into a series and subjected to SDS-PAGE, followed by immunoblot analysis using antibodies against Cas9 or phototropin (Mpphot) (see Materials and methods). Cas9-NLS (160 kDa) and Mpphot (123 kDa) were detected at positions for their expected molecular weights (closed and open arrowheads, respectively). The membranes were stained with Coomassie Brilliant Blue (CBB) and shown below. Patterns of Mpphot detection and CBB staining indicate the loading of equivalent amount of proteins between the two samples. (PDF) [file pone.0205117.s008.pdf]

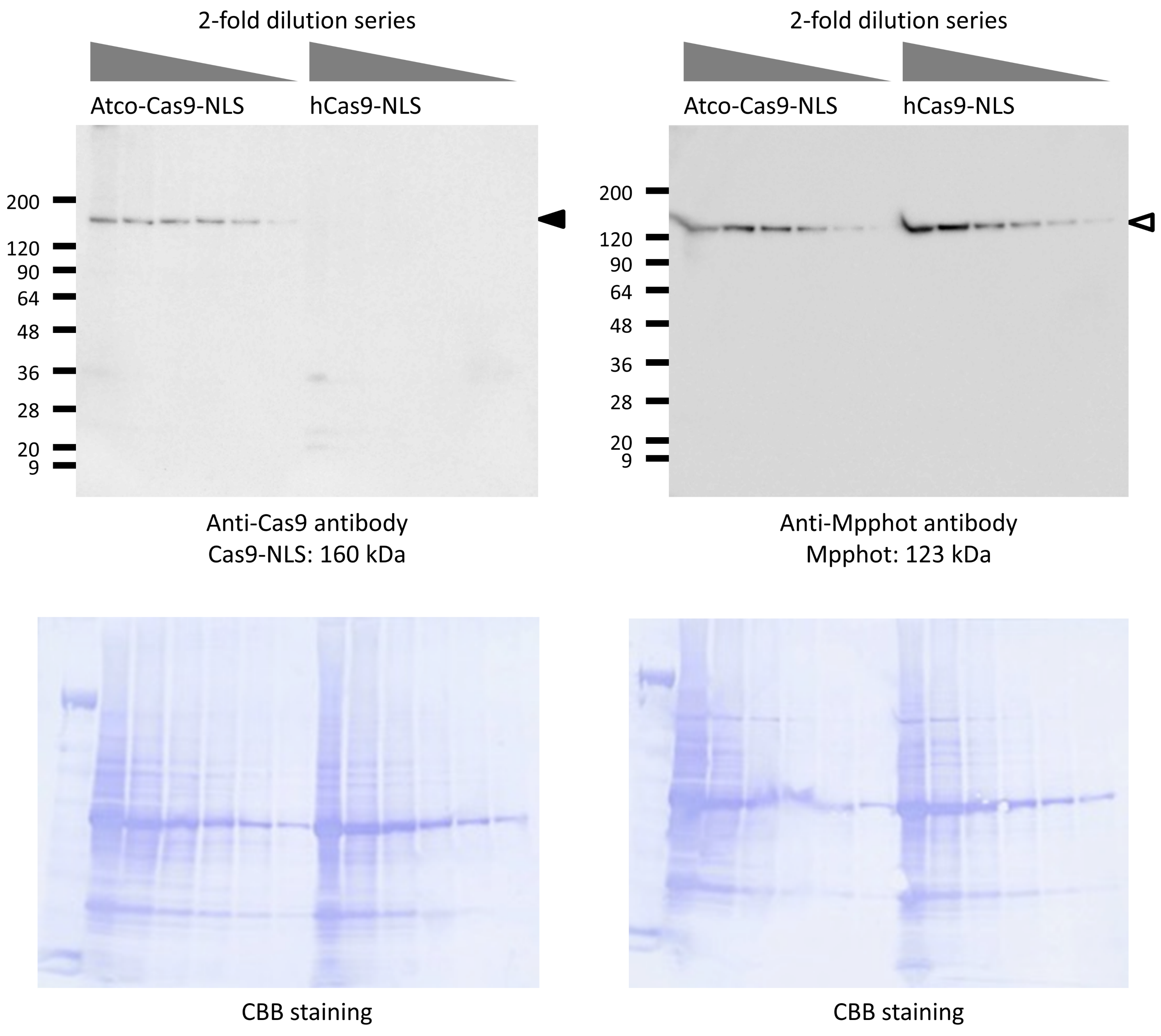
2-fold dilution series
Atco-Cas9-NLS
hCas9-NLS
200
120
90
64
48
36
28
20
9
Anti-Cas9 antibody
Cas9-NLS: 160 kDa
2-fold dilution series
Atco-Cas9-NLS
hCas9-NLS
200
120
90
64
48
36
28
20
9
Anti-Mpphot antibody
Mpphot: 123 kDa
CBB staining
CBB staining
