# Supplementary material for: Efficient CRISPR/Cas9-based genome editing and its application to conditional genetic analysis in Marchantia polymorpha
Source: PLoS One. 2018 Oct 31;13(10):e0205117. doi: 10.1371/journal.pone.0205117 (PMC6209168; doi:10.1371/journal.pone.0205117)
Supplement: S9 Fig — (A) Alignment of gDNA sequence of #13. Microhomology was highlighted with yellow and blue. (B) Schematic putative repair pathway in the mutant # 13 in pMpGE011_ARF1_1. The sequence was the same as S4 Fig. (PDF) [file pone.0205117.s009.pdf]

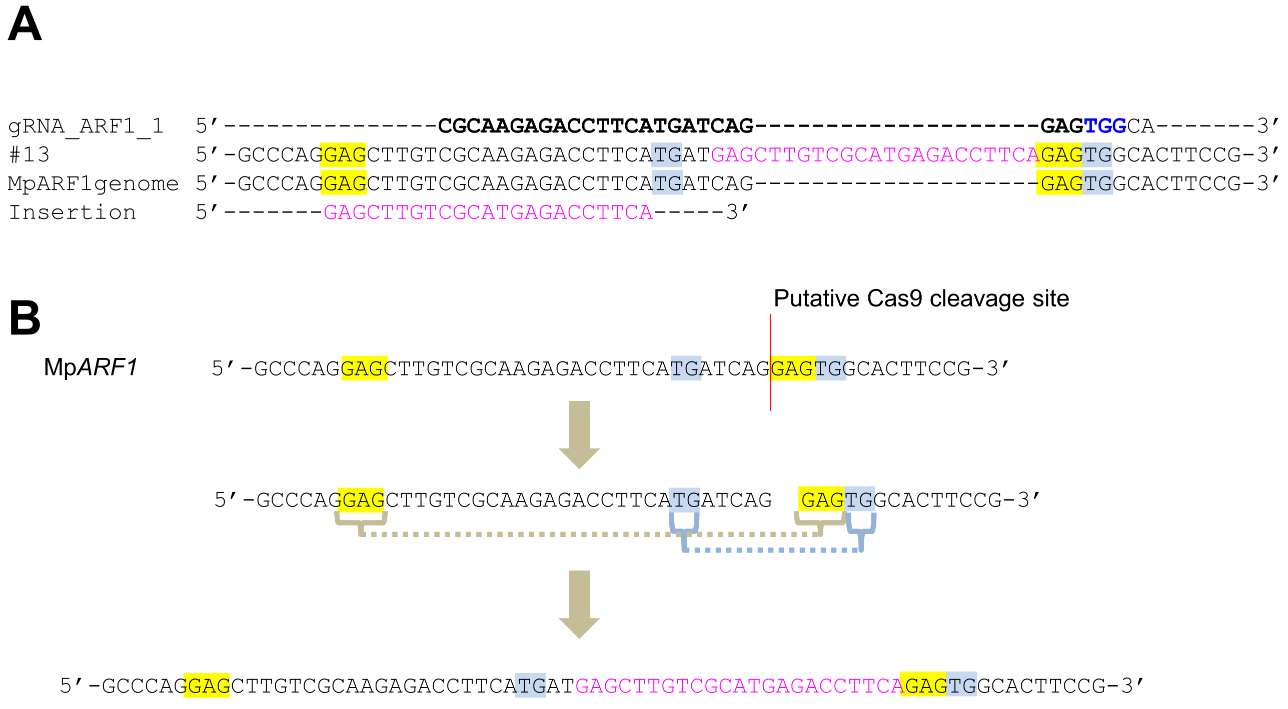
A
gRNA_ARF1_1 5′-----------------CGCAAGAGACCTTCATGATCAG-----------------------GAGTGGCA--------3′
#13 5′-GCCCAGGAGCTTGTCGCAAGAGACCTTCATGATGAGCTTGTCGCATGAGACCTTCAGAGTGGCACTTCCG-3′
MpARF1genome 5′-GCCCAGGAGCTTGTCGCAAGAGACCTTCATGATCAG-----------------------GAGTGGCACTTCCG-3′
Insertion 5′--------GAGCTTGTCGCATGAGACCTTCA-----3′
B
Putative Cas9 cleavage site
MpARF1
5′-GCCCAGGAGCTTGTCGCAAGAGACCTTCATGATCAGGAGTGGCACTTCCG-3′
5′-GCCCAGGAGCTTGTCGCAAGAGACCTTCATGATCAG GAGTGGCACTTCCG-3′
5′-GCCCAGGAGCTTGTCGCAAGAGACCTTCATGATGAGCTTGTCGCATGAGACCTTCAGAGTGGCACTTCCG-3′
