# Supplementary material for: Efficient CRISPR/Cas9-based genome editing and its application to conditional genetic analysis in Marchantia polymorpha
Source: PLoS One. 2018 Oct 31;13(10):e0205117. doi: 10.1371/journal.pone.0205117 (PMC6209168; doi:10.1371/journal.pone.0205117)
Supplement: S1 Table — (PDF) [file pone.0205117.s010.pdf]

Purpose	Name	Sequence	Related figures
Atco-Cas9 cloning	cacc_AtCas9_F	5'-CACCATGGATAAGAAGTACTCTATCGG-3'	Fig 1
Atco-Cas9 cloning	Pea3Ter_R	5'-AAGCCTATACTGTACTTAACTTGATT-3'	
Gateway cloning site amplification	Infusion_GW_A51_F	5'-GTGGTTGATAACAGCGGTTGACTAGAGTTATCA-3'	Fig 2
Gateway cloning site amplification	Infusion_GW_A51_R	5'-ATTCGAGCTCTAAGCCTCTAAGCGCTGTTATCA-3'	
MpU6-1 cloning	Mp-U6_38003_F	5'-CACCTATTCATTCAAAAGAGATTTTTAAAGATC-3'	
MpU6-1 cloning	Mp-U6_38003_R	5'-GAGAGGCTGGGTGCAAC-3'	
ccdB site amplification	OE-MpU6-CmRccdB-F2	5'-GCAGAGTTGCACCCAGCCTCTCgagctcATTAGGCACCCCAGGCTTT-3'	
ccdB site amplification	gRNA-R3	5'-tagAAAAAAAGCACCGACTCGGTG-3'	
pMpGE_En02 construction	BsaI-Sp-sgRNA_F	5'-GACCGAGAGAGGGTCTCAGTTTTAGAGCTAGAAAT-3'	
pMpGE_En02 construction	gRNA_R	5'- GTGGCACCGAGTCGGTGCTTTTTTTCTA-3'	
pMpGE_En02 construction	MpU6-1_500_F	5'-GTAACGTGAGACTACTAC-3'	
pMpGE_En02 construction	BsaI_MpU6_1R	5'-GACCCTCTCTCGGTCTCCGAGAGGCTGGGTGCAAC-3'	
pMpGE_En03 construction	Mp_oligo6BsaI_Gf	5'-TCTCGGGAGACCGAGAGAGGGTCTCA-3'	
pMpGE_En03 construction	Mp_oligo6BsaI_Gr	5'-AAACTGAGACCCTCTCTCGGTCTCCC-3'	
pMpGE013 construction	Mp_oligo5AarI_Gf	5'-TCTCGAAATGCAGGTGATGACTCACCTGCATAA-3'	
pMpGE014 construction	Mp_oligo5AarI_Gr	5'-AAACTTATGCAGGTGAGTCATCACCTGCATTTC-3'	
ARF1_1 gRNA	ARF1_sgRNA_F	5'-GCACCCAGCCTCTCGAGACCTTCATGATCAGGAGGTTTTAGAGCTAGAA-3'	Fig 3 and S4 Fig
	ARF1_sgRNA_R	5'-TTCTAGCTCTAAAACCTCCTGATCATGAAGGTCTCGAGAGGCTGGGTGC-3'	
NOP1_1 gRNA for pMpGE_En01	NOP1_sgRNA_F	5'-GCACCCAGCCTCTCGATAGTCTTTGTGAGAGAATGTTTTAGAGCTAGAA-3'	Fig 3
	NOP1_sgRNA_R	5'-TTCTAGCTCTAAAACATTCTCTCACAAAGACTATCGAGAGGCTGGGTGC-3'	
NOP1_1 gRNA for pMpGE_En02	Mp_oligo2_F	5'-TCTCGATAGTCTTTGTGAGAGAAT-3'	S4 Fig
	Mp_oligo2_R	5'-AAACATTCTCTCACAAAGACTATC-3'	
Mp*ARF1* genotyping	ARF1_Seq_F3	5'-GCCGATGTGCATATACCCAGCTATCCCAGT-3'	Fig 1 and Fig 3
	ARF1_Seq_R3	5'-ATGTTATATCCTCGGTTGATTCTCGTACGA-3'	
Mp*NOP1* genotyping	CRISPR_NOP1_F	5'-ATGGAGCAAGTGCGGTTGAGGGCTCTCG-3'	Fig 1 and Fig 3
	CRISPR_NOP1_R	5'-CGTGAGGTGACGATGCCAGTCCGACCAG-3'	
Mp*ARF1* off targets genotyping_OT1	ARF1_1_OT1_F2	5'-CAAACAATGACAGGTGAACAGCG-3'	Table 1
	ARF1_1_OT1_R2	5'-CTTCAATGAGCGTTAGTGCGAGC-3'	
Mp*ARF1* off targets genotyping_OT2	ARF1_1_OT2_F	5'-TCTCGTGACTCGATCAAGATGGG-3'	
	ARF1_1_OT2_R	5'-TTCACTCCCCTCGGCATGGTTTC-3'	
Mp*ARF1* off targets genotyping_OT3	ARF1_1_OT3_F	5'-CATCCACTCCCAGCTGTACCATC-3'	
	ARF1_1_OT3_R	5'-TCCATGGGCCTTAGATTAGGAGG-3'	
Mp*NOP1* off targets genotyping_OT1	NOP1_1_OT1_F	5'-CTCAACCATTGAACCAGCGTCGG-3'	Table 1
	NOP1_1_OT1_R	5'-TTTGTCCAGTTAGGCTGCTCATC-3'	
Mp*NOP1* off targets genotyping_OT2	NOP1_1_OT2_F	5'-TCGGGGCCTGCTCATCGGGCTCC-3'	
	NOP1_1_OT2_R	5'-GCTCAAGCTATGCCGAGGTCGTC-3'	
Mp*NOP1* off targets genotyping_OT3	NOP1_1_OT3_F	5'-TGGATTCGCCGCGTCCTCGTTCC-3'	
	NOP1_1_OT3_R	5'-TATTGGAATCGGCAGGACCGCGG-3'	
gRNA length assessment	NOP1-2_20ntF	5'-tctcCAAACCGGAATGAGTCAGCT-3'	Figs 4, 5 and S5 Fig
	NOP1-2_20ntR	5'-aaacAGCTGACTCATTCCGGTTTG-3'	
	NOP1-2_19ntF	5'-tctcAAACCGGAATGAGTCAGCT-3'	
	NOP1-2_19ntR	5'-aaacAGCTGACTCATTCCGGTTT-3'	
	NOP1-2_18ntF	5'-tctcAACCGGAATGAGTCAGCT-3'	
	NOP1-2_18ntR	5'-aaacAGCTGACTCATTCCGGTT-3'	
	NOP1-2_17ntF	5'-tctcACCGGAATGAGTCAGCT-3'	
	NOP1-2_17ntR	5'-aaacAGCTGACTCATTCCGGT-3'	
	NOP1-2_16ntF	5'-tctcCCGGAATGAGTCAGCT-3'	
	NOP1-2_16ntR	5'-aaacAGCTGACTCATTCCGG-3'	
For addition of extra intial G	NOP1-2_20ntFg	5'-ctcgCAAACCGGAATGAGTCAGCT-3'	
	NOP1-2_19ntFg	5'-ctcgAAACCGGAATGAGTCAGCT-3'	
	NOP1-2_18ntFg	5'-ctcgAACCGGAATGAGTCAGCT-3'	
	NOP1-2_17ntFg	5'-ctcgACCGGAATGAGTCAGCT-3'	
	NOP1-2_16ntFg	5'-ctcgCCGGAATGAGTCAGCT-3'	
Larger deletion analysis genotyping	NOP1_05_F	5'-CCTCATGGATTTTATCGC-3'	Fig 6 and S6 Fig
	NOP1_1k_R	5'-ACGATGGCACCAGCACT-3'	
	NOP1_2k_R	5'-TCCGACCTTTTGAAACAC-3'	
	NOP1_5k_R	5'-CCAACAATATTCAGCGAC-3'	
For ds oligo DNA	NOP1_3_F	5'-CTCGATTAAGAGTGGAAGTTGCTT-3'	
	NOP1_3_R	5'-AAACAAGCAACTTCCACTCTTAAT-3'	
	NOP1_4_F	5'-CTCGAGCTTCTCCAAGTTCTGGTC-3'	
	NOP1_4_R	5'-AAACGACCAGAACTTGGAGAAGCT-3'	
	NOP1_5_F	5'-CTCGCACGTTCACACGGCCATGGT-3'	
	NOP1_5_R	5'-AAACACCATGGCCGTGTGAACGTG-3'	
	NOP1_6_F	5'-CTCGGAAGATCAAGCATGAATCAA-3'	
	NOP1_6_R	5'-AAACTTGATTCATGCTTGATCTTC-3'	
Mp*MPK1* gRNA	sgRNA_Bsa_MpMPK1_ex1t_F	5'-tctcGGGGCCTACGGCATTGTC-3'	Fig 7
Mp*MPK1* gRNA	sgRNA_Bsa_MpMPK1_ex1t_R	5'-aaacGACAATGCCGTAGGCCCC-3'	
Mp*MPK1* CRISPR genotyping and set 3	MpMPK1_-549F	5'-GGTCGAACGCACCCTTGCAGC-3'	
Mp*MPK1* CRISPR genotyping	MpMPK1_g540R	5'-CCAAAGTAGAGGCCATGCATGTG-3'	
Mp*MPK1* cDNA	MpMPK1_1F_TOPO	5'-caccATGGATTCCGCAGCAGCTGCCG-3'	
Mp*MPK1* cDNA and set 1	MpMPK1_c1131R_STP	5'-CTATTGCATCATGTCTGGTAGGGG-3'	
Sets 1 and 2	MpEF-P_seqL1	5'-CCCACTTTGGTCAGTCCTGT-3'	
Set 2	tdTomato_753R	5'-GATGACGGCCATGTTGTTG-3'	
Set 3	MpMPK1_g1010R	5'-CGAGACATCAGGGACACGGAAG-3'	
For pMpGWB337 series	ccdB_236F	5'-AAGTGGCTGATCTCAGCCACC-3'	S7 Fig
	loxP_NruI_Sac_R	5'-TTCGAGCTCTTTCGCGATAACTTCGTATAATGTATGC-3'	
	NOSt_head_R_SacI	5'-CAGCATGAGCGAGCTGATTAAG-3'	
	mCherry_CAGC_F	5'-GATCGGGGAAATTCGAGCTC-3'	
	mTurq_CAGC_F	5'-CAGCATGGTGAGCAAGGGCG-3'	
	TagRFP_CAGC_F	5'-CAGCATGGTGAGCAAGGGCGAGG-3'	
	tdTomato_CAGC_F	5'-CAGCATGGTGTCTAAGGGTGAGGAAC-3'	
	pUGW_Aor_mTurq_IF_F	5'-GTGGTTGATAACAGCATGGTGTCTAAGGGTGAGGAAC-3'	
	pUGW_Aor_mTurq_Stp_IF_R	5'-ATTCGAGCTCTAAGCCTATTTGTAAAGCTCATCCATTCCG-3'	
